# Neutrophil-specific interactome of ARHGAP25 reveals novel partners and regulatory insights

**DOI:** 10.1038/s41598-024-71002-4

**Published:** 2024-08-29

**Authors:** Péter Sasvári, Aladár Pettkó-Szandtner, Éva Wisniewski, Roland Csépányi-Kömi

**Affiliations:** 1 Department of Physiology, Semmelweis University, Tűzoltó u. 37-47., Budapest, 1085 Hungary; 2 Proteomics Research Group, Core Facility, HUN-REN Biological Research Centre, Szeged, 6726 Hungary

**Keywords:** 14-3-3 Proteins, ARHGAP25, Protein–protein interactions, RAC2, RHOG, Proteomics, Protein-protein interaction networks, Neutrophils

## Abstract

ARHGAP25, a crucial molecule in immunological processes, serves as a Rac-specific GTPase-activating protein. Its role in cell migration and phagocyte functions, affecting the outcome of complex immunological diseases such as rheumatoid arthritis, renders it a promising target for drug research. Despite its importance, our knowledge of its intracellular interactions is still limited. This study employed proteomic analysis of glutathione S-transferase (GST)-tag pulldowns and co-immunoprecipitation from neutrophilic granulocyte cell lysate, revealing 76 candidates for potential physical interactions that complement ARHGAP25’s known profile. Notably, four small GTPases (RAC2, RHOG, ARF4, and RAB27A) exhibited high affinity for ARHGAP25. The ARHGAP25–RAC2 and ARHGAP25–RHOG interactions appeared to be affected by the activation state of the small GTPases, suggesting a GTP–GDP cycle-dependent interaction. In silico dimer prediction pinpointed ARHGAP25’s GAP domain as a credible binding interface, suggesting its suitability for GTP hydrolysis. Additionally, a list of Fc receptor-related kinases, phosphatases, and three of the 14-3-3 members were identified as potential partners, with in silico predictions highlighting eight binding sites, presenting novel insight on a potential regulatory mechanism for ARHGAP25.

## Introduction

Rho-family small GTPases (smgs) regulate a broad range of cellular functions, including cell morphology, migration, and membrane trafficking, essential for immunological functions such as phagocytosis and superoxide production^1^. These molecular switches undergo a conformational change upon binding GTP, allowing their interaction with effector proteins. Slow hydrolysis of GTP reverses the active conformation, leading to the termination of intracellular signaling. Currently, 65 human proteins are known to carry a specific, GTPase-activating (GAP) domain, capable of significantly enhancing the GTP-hydrolysis of Rho-family smgs, thereby contributing to signal-termination^2^. These RhoGAPs have diverse domain structures, varied tissue expression, and intracellular localization. Along with their variability, the precise functional roles of only a few RhoGAPs have been elucidated, with a limited understanding of their potential protein interactions^3,4^.

One of them is ARHGAP25, which contains a Rac-specific GAP domain surrounded by an N-terminal pleckstrin-homology (PH) domain and a C-terminal superhelical (coiled-coil) region, linked together by disorganized sequences^4,5^.

Previous studies mainly focused on ARHGAP25’s functions in leukocytes: it has been shown to act as a negative regulator of phagocytosis by targeting RAC1 and affect peripheral B-cell development and antigen-response^6–8^. In a cell-free superoxide-generating system used by Lorincz et al., the addition of soluble ARHGAP25 decreased superoxide production. At the same time, the depletion of membrane-associated ARHGAP25 increased superoxide production by affecting RAC activation, highlighting its crucial role in regulating reactive oxygen species production^9^. Moreover, high expression of ARHGAP25 in synoviocytes during autoantibody-induced arthritis has been demonstrated to play an essential role in the production of cytokines during inflammation^10^.

Participating in tightly regulated processes, ARHGAP25 is also expected to undergo regulation itself. Phosphorylation as a means for post-translational regulation was first proposed by Wang et al. when they reported the serine residue at position 362 (S362) as a critical phosphosite affecting ARHGAP25’s GAP activity and its role in hematopoietic stem cell and progenitor cell mobilization from bone marrow^6^. Subsequent work by Wisniewski et al. revealed two additional phosphosites (S378-379 and S487) as key regulatory sites, influencing in vitro GAP activity as well as in vivo superoxide production, actin depolymerization, and phagocytosis^11^. Nonetheless, our understanding of the upstream actors and pathways involved in this regulation remains unclear.

Contrary to its specific abundance in leukocytes, ARHGAP25’s involvement in various cancer types has emerged in recent years. It appears to function as a tumor suppressor in pancreatic adenocarcinoma, colorectal, lung, and breast cancer, allegedly regulating the WNT/β-catenin and Akt/mTOR pathways^12–15^. Even though the direct evidence of ARHGAP25 playing a relevant role in non-hematopoietic cells during pathophysiological processes is still insufficient, the possibility of it taking part in other pathways might open new avenues for cancer treatment.

Here, by selecting an approach to provide a more physiological environment using human neutrophilic cells, we demonstrate that ARHGAP25 is co-eluted with various previously unrecognized protein partners. Out of the 90 candidates showing great affinity towards GST-ARHGAP25, co-immunoprecipitation (co-IP) with the endogenous form confirmed 34 with high, 26 with satisfactory, and 16 with low credibility. Four small GTPases (RAC2, RHOG, ARF4, RAB27A) are detected among the potential candidates, with ARHGAP25 favoring the active (GTP-bound) form of RAC2 and RHOG, but not ARF4 or RAB27A. In silico dimer prediction for ARHGAP25–RAC2 and ARHGAP25–RHOG has provided high-quality (ipTM > 0.85) simulations for direct interaction involving the GAP domain, suggesting a potential role for ARHGAP25 in their GTP–GDP cycle. A sizeable number of kinases, phosphatases, and three specific members of the 14-3-3 protein family are identified as potential ARHGAP25 binding partners. Our analysis also pinpoints eight potential phosphorylation-dependent binding sites on ARHGAP25, proposing novel mechanisms and pathways for the phosphorylation-dependent intracellular regulation of this protein.

## Results

### GST-ARHGAP25 pulldown demonstrates significant protein enrichment from neutrophilic granulocyte cell lysate compared to GST control

**Fig. 1:**
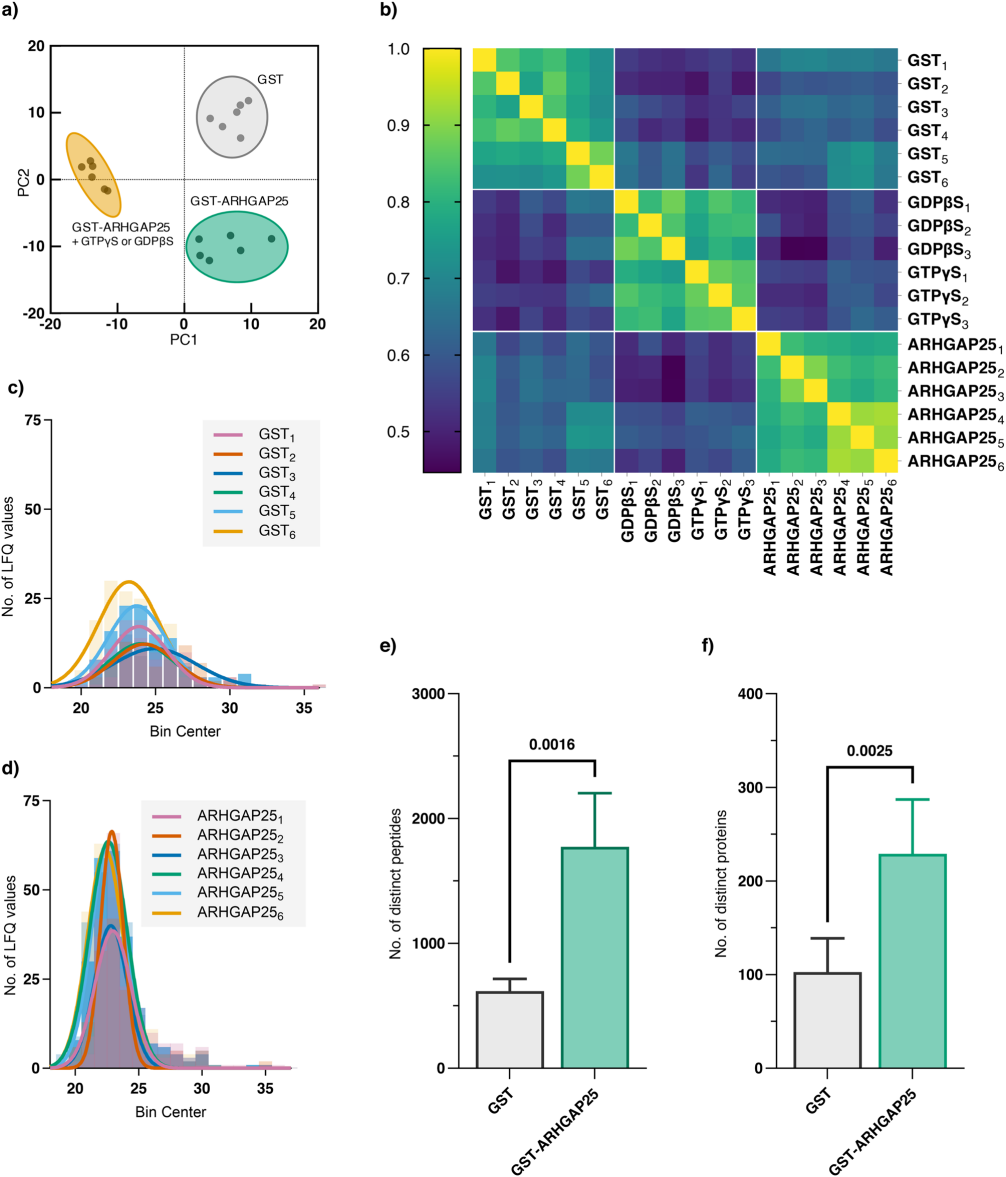
Mass spectrometric analysis of GST-ARHGAP25 pulldown from neutrophil cell lysate identified numerous co-eluted proteins. Pulldown assay was performed with GST-tagged recombinant ARHGAP25 and GST control from human neutrophil cell lysate and analyzed by LC–MS/MS; subsequent quality checks were performed. (**a**) PCA representation of sample clustering based on used bait (GST and GST-ARHGAP25, colored grey and green, respectively) from intact neutrophilic cell lysate and separation of GST-ARHGAP25 pulldowns from pretreated cell lysate (colored orange). The samples clustered into three groups with no sign of batch effect. (**b**) Pearson correlation matrix of all samples, shown as a heatmap. Samples showed higher correlations to the ones in their own group than the others. (**c**) Histogram of unimputed LFQ values of GST samples. (**d**) Histogram of unimputed LFQ values of GST-ARHGAP25 samples. The values follow a Gaussian distribution, making them eligible for the following statistical comparisons. (**e**) Comparison of detected distinct peptides in GST (colored grey) and GST-ARHGAP25 samples (colored green). (**f**) Comparison of detected distinct peptides in GST (colored grey) and GST-ARHGAP25 samples (colored green). A significant increase was detected in the case of GST-ARHGAP25 pulldown. In (**e**–**f**), data are presented as mean ± SD (n = 6). Employed statistics: unpaired Student t-test. *p* values are presented numerically and considered significant below 0.05.

To isolate the potential interaction partners of ARHGAP25, GST-fused, recombinant ARHGAP25 was incubated with either intact or nucleoside derivate-treated neutrophilic cell lysate, and the final eluates were processed by mass spectrometry. GDPβS and GTPγS treatment served as a means for altering the GTP/GDP levels of the related proteins in the cell lysate. Pulldowns using GST alone were also conducted to exclude nonspecific binding proteins. Before evaluating the results, we conducted quality control checks on the acquired mass spectrometry (MS) data. The label-free quantification intensity (LFQ) values of proteomic data, calculated by the FragPipe software, were utilized to generate a Principal Component Analysis (PCA) plot and a Pearson correlation matrix (Fig. 1a,b). Data visualization by the PCA plot demonstrated the formation of distinct clusters: the control samples, in which the pulldown experiment was carried out with GST alone separated from the GST-ARHGAP25 pulldown samples. Similarly, the correlation matrix exhibited a comparable separation pattern, with pulldowns from the same protein pools (intact cell lysates, GDPβS, and GTPγS loaded cell lysates) displaying higher Pearson correlation values than those between different pools. The frequency distribution of logarithmic LFQ values followed the Gaussian distribution, making it eligible for statistical analysis (Fig. 1c,d).

The number of detected distinct peptides and proteins identified by the FragPipe software in each sample (before imputation) was counted and compared. All GST-ARHGAP25 pulldown samples consistently yielded peptide and protein counts higher than in the GST control samples, demonstrating a significant enrichment (Fig. 1e,f). Out of the 775 identified proteins, 71 (9.16%) were detected solely in GST eluate and 471 (60.77%) solely in GST-ARHGAP25 eluates, while 233 (30.07%) overlapped. These data suggest that GST-ARHGAP25 formed direct or indirect physical associations with numerous proteins with bonds strong enough to make their isolation possible. The search results are available in Supplementary Table S1.

### In human neutrophilic granulocytes, 76 potential protein partners of ARHGAP25 were identified

**Fig. 2:**
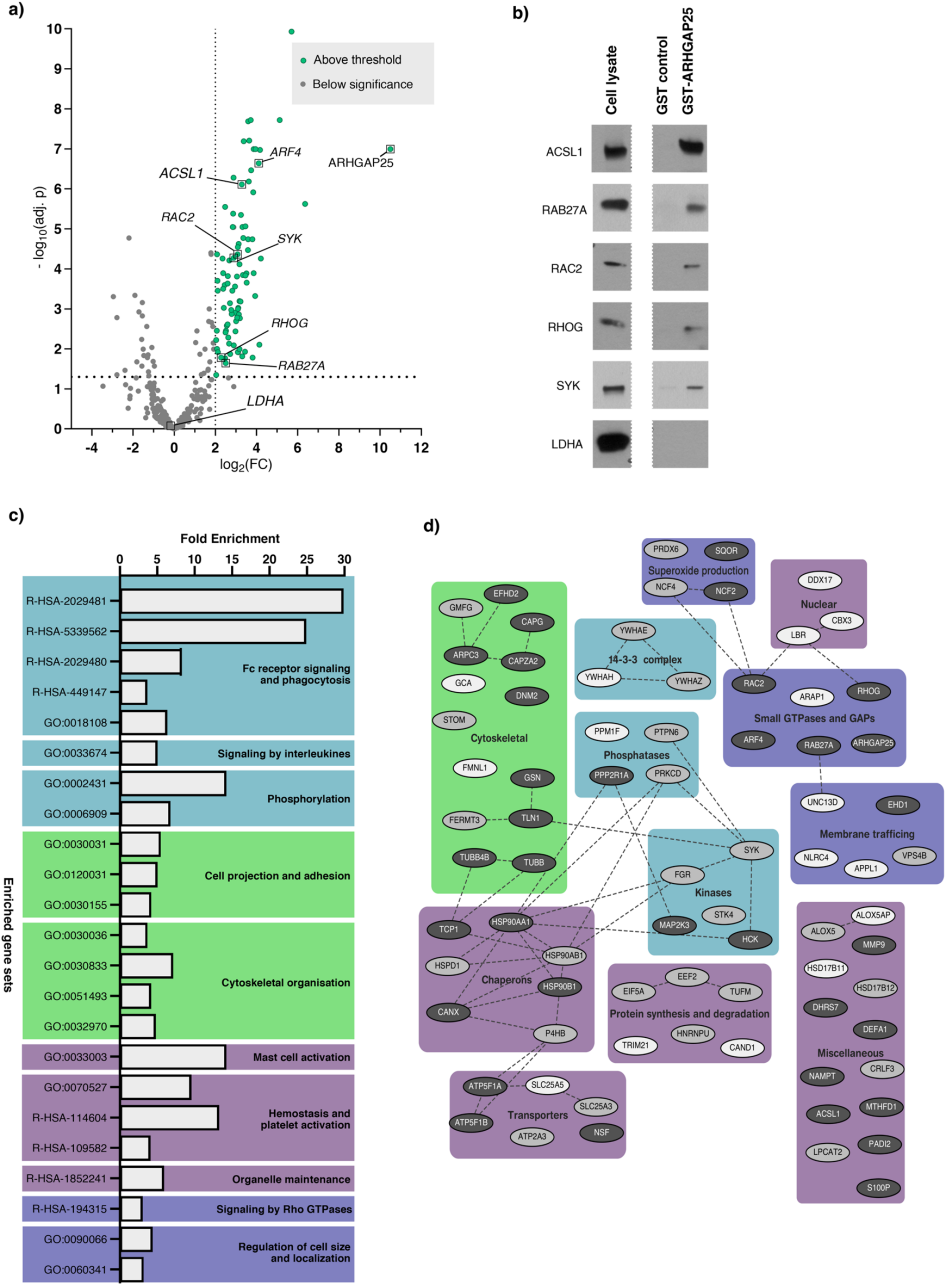
Identification of ARHGAP25’s interactome in neutrophilic granulocytes. Out of the identified proteins, a list of potential candidates of interaction partners was selected and further supported by co-immunoprecipitation assays from neutrophil cell lysates. (**a**) Volcano plot of the GST-pulldown-derived proteomic data. Employed statistics: multiple t-tests with Benjamini–Hochberg correction (n = 6); significance thresholds are marked by dotted lines. ARHGAP25’s potential partners are colored green. Gray dots represent proteins below the threshold. Ninety proteins showed a significant preference towards GST-ARHGAP25. (**b**) Western Blot validation of the proteomic analysis. Six proteins were selected to check their presence in the samples. Representative images reinsured the presence of ACSL1, SYK, RAB27A, RHOG, and RAC2, and the absence of LDHA. (**c**) Functional enrichment of ‘GO: Biological Function’ and ‘Reactome’ gene sets of identified protein partners. Gene sets with redundant or overlapping themes are grouped and colored differently for clarity. (**d**) STRING (‘Search Tool for Retrieval of Interacting Genes/Proteins’) analysis between identified partners of ARHGAP25. Based on molecular function and localization, proteins are separated into different groups. Lines represent documented interactions between proteins extracted from the STRING database.

To depict the relative protein abundance between GST-ARHGAP25 pulldown samples obtained from intact cell lysate and the GST pulldown control samples, a heatmap of LFQ values was constructed after Z-score normalization. Both the samples (represented by columns) and the proteins (represented by rows) were subjected to hierarchical clustering (Supplementary Fig. S1), leading to the formation of distinct sample clusters that corresponded to their respective experimental group (GST control and GST-ARHGAP25). The proteins were divided into three main clusters: (i) proteins with varying Z-scores across the samples, (ii) proteins displaying higher Z-scores in the GST-ARHGAP25 samples, and (iii) proteins exhibiting higher Z-scores in the control samples (Supplementary Fig. S1). Subsequent statistical analysis, employing multiple t-tests with Benjamini–Hochberg correction, identified 90 proteins enriched in GST-ARHGAP25 eluates (Fig. 2a, colored green). Among these proteins, ARHGAP25 exhibited the highest average fold change (FC) value (10.5; colored orange in Fig. 2a). Next, co-immunoprecipitation with polyclonal anti-ARHGAP25 antibody and control IgG antibody were carried out, and the relative abundances of the previously selected 90 proteins were measured by mass spectrometry. Out of the 90 candidates, 13 were excluded due to too low detection rate, while 76 (excluding ARHGAP25) were reassured by the results. Each candidate was assigned a confidence score (‘high,’ ‘moderate,’ or ‘unlikely’) based on the detection rate of the protein and an enrichment score (‘high’ or ‘low’) based on the fold change values between the two groups. The potential interactions were categorized into three groups according to their assigned confidence and enrichment scores: 16 interactions were rated ‘low credibility’ (meaning moderate confidence and low enrichment), 26 received a ‘satisfactory’ (having either high confidence and low enrichment or moderate confidence and high enrichment), and 34 received a ‘high credibility’ rating (high confidence and high enrichment). The list of proteins enriched significantly in GST-ARHGAP25 pulldown samples compared to GST and their corresponding co-IP scores are available in Supplementary Table S2.

### Western Blot analysis carried out from pulldown eluates supports the validity of our proteomic results

To assess the accuracy of the MS data, we have chosen six proteins from the pulldown measurements with different fold changes and p values (Fig. 2a; Supplementary Fig. S1). ACSL1, RAC2, RHOG, RAB27A, and SYK were selected from the significant candidates (upper right quadrant in Fig. 2a),and LDHA were chosen from the proteins with low FC and high adjusted (adj.) *p*-value (lower left). Representative Western blots are shown in Fig. 2b. Proteins having received ‘high credibility’ (ACSL1, RAC2, RHOG, RAB27A) and ‘satisfying’ (SYK) scores were consistently detected by antibodies and demonstrated decent enrichment. LDHA, which was undetectable by MS, was also undetectable by Western Blot experiments. Uncropped images are collected in Supplementary Fig. S2.

### Biological functions associated with the current candidate partners align with ARHGAP25’s known functions

Given the substantial number of associations, we aimed to investigate whether the identified set of proteins shared common intracellular functions or pathways. To address this, a functional enrichment analysis was conducted, by using the shinyGO: web server. Applying “Gene Ontology (GO) biological process” and “Reactome” data sources revealed overrepresented gene sets that primarily clustered around 10 key themes (Fig. 2c). First, we identified a hub of 5 gene sets linked to the Fc receptor signaling pathway, thereby strongly associated with phagocytosis. The overrepresented proteins were also key players in interleukine-mediated signaling and phosphorylation. A substantial number of terms related to cell projection and cytoskeletal organization emerged, as well as ones connected to GTPase-mediated signaling.

Moreover, gene sets related to mast cell and platelet activation, hemostasis, and organell maintenance were also overrepresented. These results suggest that our identified interactome nicely complements our current knowledge about ARHGAP25’s proposed neutrophil functions and provides new insights.

The STRING (‘Search Tool for Retrieval of Interacting Genes/Proteins’) platform was employed to gather all experimentally determined interactions between enriched proteins, which were then represented as a network in Fig. 2d. This resulted in a total of 49 associations involving 45 interactors, which could imply the presence of eluted multimers in the samples. Besides the expected presence of cytoskeletal and GTPase-related proteins, many kinases, phosphatases, and chaperones were identified. Members of the 14-3-3 family were also present, and proteins connected to membrane trafficking, protein synthesis, and degradation. The acquired network, visualized based on cellular localization and molecular function, provides insight into the significant candidates.

### Formerly unreported small GTPases emerged as potential interaction partners for ARHGAP25

Among the 76 proteins, a few small GTPases enriched in GTP-ARHGAP25 eluates. RAC2, a member of the Rac subclass that is most prominent in neutrophilic granulocytes, exhibited an average log_2_FC of 3.08 and 2.57 in the pulldown and co-IP studies, respectively. Analyzing the peptides identified by MS, four unique sequences supported the presence of the RAC2 isoform (refer to Supplementary Table S1). Interestingly, another member of the Rac subfamily, RHOG, was detected in 4 pulldowns (67%) and 5 (100%) co-IP samples and exhibited a log_2_FC of 2.28 and 2.75 in the pulldown and co-IP studies, respectively. Aligned with previous results^8^, we did not find evidence that any Rho or Cdc42 subfamily members eluted with GST-ARHGAP25. Surprisingly, ARF4 and RAB27A were among the potential protein interactions as well.

### GTPγS or GDBβS loading of small GTPases did not have a prominent effect on the ARHGAP25 proteome

**Fig. 3:**
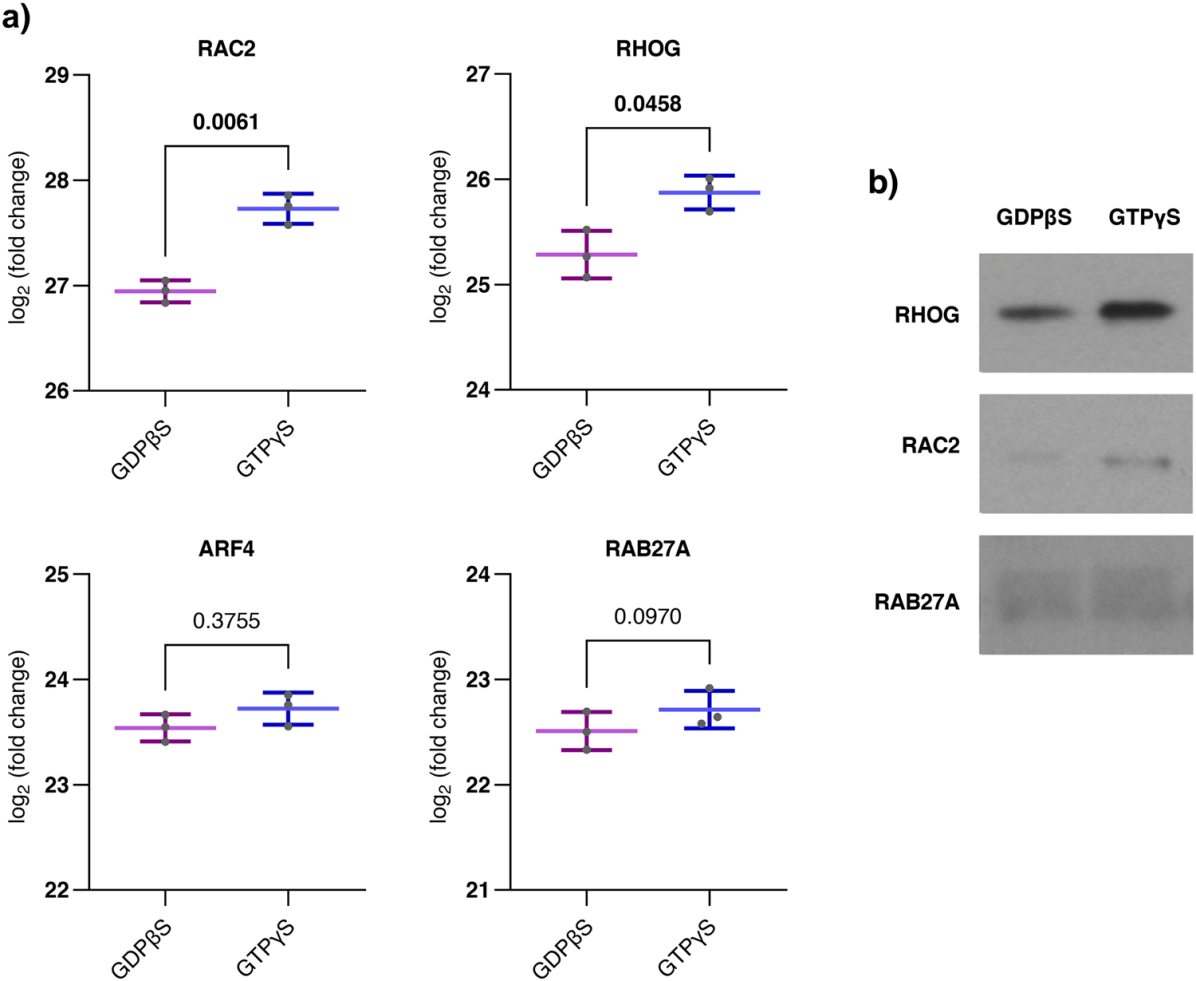
Assessment of the effect of GTPγS and GDPβS loading of cell lysate before pulldown. To investigate the impact of the GTP/GDP cycle of small GTPases on their affinity to ARHGAP25, we performed GST-pulldowns on samples pretreated with GTPγS and GDPβS. (**a**) Comparison of LFQ values of small GTPases pulled down with ARHGAP25 following GTPγS and GDPβS loading of the neutrophilic cell lysate. Employed statistics: paired t-tests (n = 3). Data are presented as mean ± SD. *p* values are presented numerically; bold values are considered significant (*p* < 0.05). LFQ values RAC2 and RHOG were significantly elevated in GTPγS samples, implying a GTP/GDP dependent effect on their affinity towards ARHGAP25. ARF4 and RAB27A did not show any significant difference. (**b**) Representative Western Blot of RHOG, RAC2 and RAB27A protein levels in GST-ARHGAP25 pulldown samples following GTPγS- and GDPβS-loading of the neutrophilic cell lysate. The difference in the band intensities supported our proteomic results.

As we were curious to see the effect of GTP/GDP on formerly detected interactions, we carried out GTPγS and GDBβS loading of the cell lysate before incubation with the beads. During the loading process, the ratio of active (GTPγS-bound) and inactive (GDPβS-bound) is shifted in favor of either state, allowing us to investigate the effect of the DTP/GDP cycle on the interactome. When we examined the four identified small G-proteins individually, we noticed different LFQ values after GTPγS loading compared to GDPβS in the case of RAC2 (log_2_ΔFC = 0.785) and RHOG (log_2_ΔFC = 0.589). In contrast, ARF4 and RAB27A showed no change (log_2_ΔFC < 0.2) (Fig. 3a). Western blot experiments also confirmed the previous results in the case of RAC2 and RHOG. (Fig. 3b). These data might suggest ARHGAP25’s preference towards the active (GTP-bound) form of RAC2 and RHOG, which aligns with the GAP domain’s established specificity for the Rho family. However, the binding seems to be independent of the GDP/GTP-bound state in the case of ARF4 and RAB27a.

### In silico multimer prediction highlights potential direct protein interaction partners from the interactome pool

**Fig. 4:**
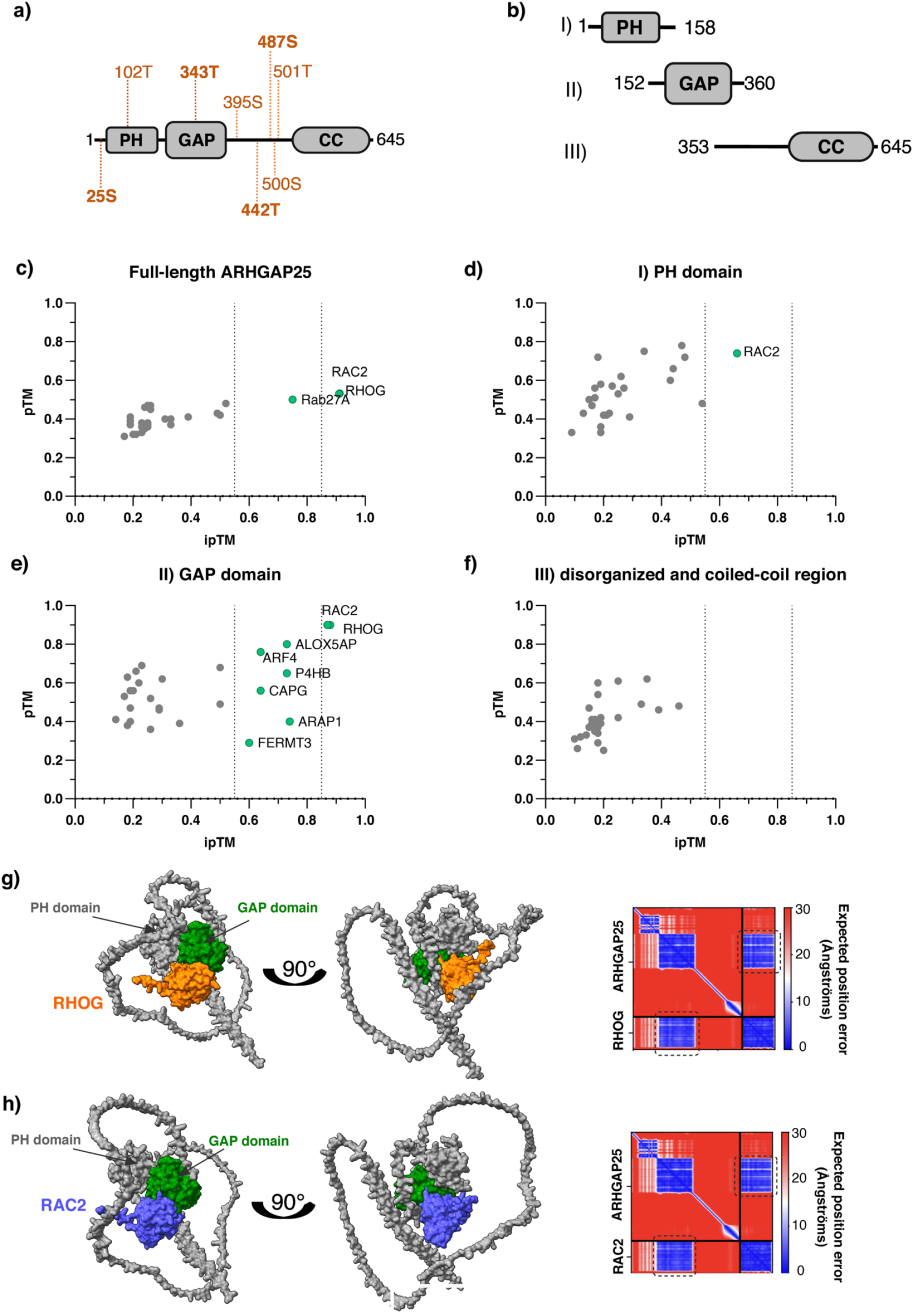
In silico experiments for highlighting potential direct interactions with ARHGAP25. Due to the vast number of potential candidates, we resorted to computer predictions to identify candidates for direct interactions. (**a**) Graphical representation of full-length canonical ARHGAP25 fragments used for dimer prediction in (**c**), along with its domain structure PH: pleckstrin homology domain, GAP domain, CC: coiled-coil region in grey and potential binding sites for 14–3-3 proteins in orange. (**b**) Graphical representation of truncated ARHGAP25 fragments used for dimer prediction in (**d**–**f**). (**c**) In silico dimer prediction between full-length ARHGAP25 and proteins identified in our study. (**d**) In silico dimer prediction between the PH domain of ARHGAP25 and proteins identified in our study. (**e**) In silico dimer prediction between the GAP domain of ARHGAP25 and proteins identified in our study. (**f**) In silico dimer prediction between the coiled-coil region of ARHGAP25 and proteins identified in our study. In (**c**–**f**), the cut-off threshold (ipTM = 0.55) and the threshold for distinguishing highly credible (ipTM > 0.85) predictions are marked by the dotted lines. Predicted dimers with ipTM > 0.55 are shown in green. Several predictions performed above the cut-off value and simulations for RHOG and RAC2 were the most reliable and, thus, eligible for further assessment. (**g**) Predicted model of RHOG (orange) and ARHGAP25 (grey; the GAP domain is highlighted in green) along with its predicted aligned error (PAE) plot. h) Predicted model of RAC2 (purple) and ARHGAP25 (grey; the GAP domain is highlighted in green) along with its predicted aligned error (PAE) plot. In both cases, the small GTPase is positioned closest to the GAP domain, where the predicted position errors were the lowest (marked with a grey dotted rectangle).

Due to the high number of significant candidates and the overrepresentation of protein associations among them, we aimed to highlight proteins that are the most probable direct potential partners for ARHGAP25. We employed AlphaFold Multimer prediction to identify credible direct binary interactions between ARHGAP25 and the candidates. Based on fold change values, intracellular localization, and protein function, we selected 28 identified proteins to simulate protein-complex formation (Supplementary Table S3). We used full-length and truncated sequences of ARHGAP25 and full-length candidate sequences (Fig. 4a,b). Ten predictions were run for each dimer, and the best prediction was determined based on the estimate of the TM-score (pTM) and intracellular TM-score (ipTM) obtained from a pairwise error prediction calculated as a linear projection from the final pair representation^16,17^. Following the guidelines of O’Reilly et al., all predictions with ipTM < 0.55 were ignored, and a cut-off value of ipTM = 0.85 was used to select the most credible predictions^18^. To eliminate the distorting effect of ARHGAP25’s size and unorganized sequence regions, we screened the exact interactions between three truncated fragments of ARHGAP25, each containing one domain region (Fig. 4c–f). The program predicted three dimers with the ful-length ARHGAP25 (RAC2, RHOG and RAB27A), eight dimers with the GAP domain (ALOX5AP, P4HB, ARAP1, ARF4, CAPG, FERMT3, RAC2 and RHOG), one dimer with the PH domain (RAC2) and no dimers with the disorganized and coiled-coil region above 0.55 ipTM score. (Fig. 4c–f). As only RHOG and RAC2 predictions were of the highest credibility (above 0.85 ipTM), we only focused on their predicted conformation in this study. Upon closer inspection of the predicted models, both small GTPases were positioned closest to ARHGAP25’s GAP domain (Fig. 4g,h colored green) with the lowest predicted aligned errors. As the predicted aligned error plots estimate the expected positional error for each residue in a predicted protein structure^16,17^, the GAP domain appears to be mainly responsible for the interaction (Fig. 4g,h, highlighted in a rectangle with dotted edges), while the PH domain and the coiled-coil region play a less prominent role.

### ARHGAP25 contains eight potential binding phosphosites for 14-3-3 proteins

As three members of the 14-3-3 proteins were identified as potential interaction partners, we wanted to assess the possibility of their binding to ARHGAP25 through in silico prediction. The 14-3-3-Pred web server used for the prediction of interaction was created by Madeira et al.^19^ and employs three distinct methods (the position-specific scoring matrix, support vector machines (SVM), and artificial neural network (ANN) classification) to identify potential residues for 14-3-3 binding upon phosphorylation. ARHGAP25 contains 57 serine and 29 threonine side chains, among which T102, S395, S500, and T502 scored highly according to only one method, and S25, T343, T442, and S487 received high scores across all three methods (Supplementary Table S3). The identified phosphosites are visualized in Fig. 4a.

## Discussion

With the emergence of high-throughput protein–protein interaction (PPI) research, ARHGAP25 has been implicated in several binary interactions^20–22^. However, upon closer inspection, the proposed candidates fail to align with our current understanding of ARHGAP25^23^. This phenomenon is not uncommon in the case of high-throughput experiments, so more specific approaches are still highly encouraged^24^.

We aimed to explore ARHGAP25’s potential role in protein interactions and determine a more plausible list of PPI candidates using a protein pool in which ARHGAP25 is endogenously expressed. We initially resorted to GST-tagged pulldowns as they provide an excellent signal-to-noise ratio with low nonspecific binding. Moreover, it has been previously demonstrated that our GST-ARHGAP25 protein construct acts as the endogenous form in vitro^8,11^. In our initial study, using GST-ARHGAP25 pulldown from human neutrophilic cell lysate, we isolated a substantial set of proteins and identified 90 unique candidates associated with GST-ARHGAP25. As recombinant proteins might introduce false positive results due to possible deviations in tertiary structure or post-translational modifications, we also performed co-immunoprecipitation of endogenous ARHGAP25 from human neutrophilic cell lysate. It served a dual purpose of filtering out unlikely partners and confirming the credibility of 76 previously selected interactions. We further categorized them into 3 groups based on the rate of detection and level of enrichment. The reliability of our mass spectrometry results was also validated by Western blot experiments, confirming the presence of the proteins identified in the MS report and relevant to our final conclusions.

The high number of partners could be an indicator of indirect interactions and present the possibility of multimers, a phenomenon likely arising from the intricate nature of small GTPases functioning within versatile protein complexes^25^. This idea is further supported by the functional enrichment results highlighting numerous sets of proteins acting together to carry out cellular processes, such as cytoskeletal organization, Fc receptor signaling, and phagocytosis. As several of these terms align with known functions of ARHGAP25, it provides further proof that our list depicts a highly plausible pool of interactions. A notable strength of our approach lies in the identification of protein partners with cell-specific protein expression, using a neutrophilic cell lysate. For example, our findings include the detection of two components of the soluble part of the NADPH oxidase complex (NCF2 also known as p67-phox and NCF4 also known as p40-phox), aligning with ARHGAP25’s role in modulating RAC-mediated superoxide production in neutrophils^9,10,26^. Moreover, several new promising candidates (e.g., components of the ARP2/3 complex and the cytoskeleton, transporters, membrane trafficking-related proteins, etc.) and related molecular functions (signaling by interleukins, organelle maintenance, hemostasis, platelet and mast cell activation, etc.) arose from our experiment that could become the cornerstone of future projects.

We must include that using tagged proteins as baits holds the possibility of disrupting the protein structure, which might result in an altered proteome. To eliminate the distorting effect of the GST tag and further minimize the probability of false positive results, we included co-immunoprecipitation studies. Furthermore, when comparing our findings to the fairly limited literature available, a significant correlation is shown. Müller et al. conducted extensive research on small GTPases, including many protein interactions for GAPs and GEFs^27^. The substantial overlap between their identified candidates and our protein partners supports the idea that these unexpected interactions might be a relevant, but still unexplored territory of GAP physiology. Similar overlaps could be found in three other studies examining protein interactions of ARHGAPs^28–30^. The substantial overlap between their identified candidates and our protein partners supports the idea that these unexpected interactions might be a relevant but still unexplored territory of GAP physiology.

During the detailed investigation of our results, we focused on the small GTPases among our results as they are the most likely candidates based on ARHGAP25’s GAP domain. Human neutrophils express two Rac isoforms (RAC1 and RAC2), although the ratio is shifted in favor of RAC2^31,32^. As ARHGAP25 has been solely associated with RAC1, it is the first time the RAC2 variant has been reported as a possible partner in our experiments, supported by four peptide sequences unique to RAC2. During our studies, we failed to detect any RAC1-specific unique peptides by MS. However, given the high RAC2 content in neutrophils and the fact that the two proteins share a 92% homology (as 177 out of 192 amino acids are identical), the lack of RAC1-specific peptides does not preclude its interaction with ARHGAP25. Furthermore, RHOG, one of the members of the Rac subfamily, was also identified and supported by co-IP and Western blot measurements, which have never been associated with ARHGAP25. Interestingly, other small GTPases, such as ARF4 and RAB27A, were also among the highly credible candidates. Although this might seem like an unexpected interaction, Müller et al. also found evidence of ARHGAP31-ARF4 interaction during their studies^27^. Kouchi et al. reported similar results between ARHGAP21, ARHGAP23, and Arf members^28^. Their computational studies appointed the PH domain of the GAPs as potential interaction sites. Our findings also raise the possibility that the substrate specificity of ARHGAP25 is more diverse than we initially thought.

During the GTP–GDP cycle of small GTPases, the active form prefers binding to GAPs instead of GEFs^25^, leading us to investigate the influence of GTP/GDP-binding on the interaction with ARHGAP25. Our MS and Western Blot experiments suggest that ARHGAP25 prefers the GTP-bound form of RAC2 and RHOG over their inactive, GDP-bound state, but no such preference was observed in the case of ARF4 and RAB27A. We speculate that ARHGAP25 might function as a GTPase activating protein (GAP) for RAC2 and RHOG, but not for ARF4 or RAB27A, or it takes part in a molecular complex involving active RAC2 and RHOG. Our in silico dimer prediction between ARHGAP25 and RAC2 or RHOG also complemented our initial idea, as the GAP domain plays a vital role as a highly credible interaction site. However, these findings imply solely a physical interaction between these proteins, and further experiments are needed to measure the GAP activity of ARHGAP25 on these small GTPases as well.

The phosphorylation of ARHGAP25 plays a crucial role in its regulation. In collaboration with Wang’s group, we first identified S362 as an important phosphosite that affects its GAP activity and reported other phosphosites that might play similar roles^6^. Based on these findings, we selected three serine amino acids to examine their impact on in vitro GAP activity and intracellular functions of ARHGAP25^11^. However, until this point, we could not assign these post-translational modifications to any kinases or phosphatases. This study identified five kinases (FGR, HCK, MAP2K3, STK4, SYK) and four phosphatases (PPM1F, PPP2R1A, PRKCD, PTPN6) as potential interacting partners that can serve as a base for further phosphoproteomic studies to elaborate on the pathways responsible for the phosphorylation of ARHGAP25 and its subsequent effect. Many of these proteins are key members in Fc-receptor-mediated pathways, which was also a reoccurring term in our functional enrichment analysis (refer to Supplementary Table S4). It has been shown by Wisniewski et al. that phosphorylation of ARHGAP25 can affect its GAP activity, allowing RAC to remain in the GTP-bound (active) form^11^. Additionally, as SYK-mediated RAC activation via GEFs has already been established^33^, regulation of ARHGAP25 by such upstream regulators could complement our current understanding of Fc receptor-mediated RAC activation. However, future measurements are needed to prove this connection on a cellular level.

Three members of the 14-3-3 protein family (YWHAE, YWHAZ, YWHAH) have also been identified as potential partners. Their role in cell biology is diverse due to their capability to bind to phosphorylated peptide sequences, which can lead to, in some instances, the inhibition of the target protein^34,35^. This effect has been explored in the case of SOS1, a RAS-specific GEF^36^. Furthermore, many studies on GAP PPIs detected members of the 14-3-3 family as candidates^27,28,30^. Our in silico prediction highlighted eight phosphosites on ARHGAP25 as possible interaction sites. In a previous study, one of them, S487, has already been explored as a potential modulatory site for ARHGAP25’s intracellular function^11^. Even though exploring the effect of 14-3-3 proteins on ARHGAP25 functions is out of the scope of this article, these novel findings can pave the way for a potential new mechanism for ARHGAP25 modulation.

The potential tumor-suppressing role of ARHGAP25 has recently been connected to various intracellular pathways. In some instances, it is argued to exert this effect via the regulation of RAC1-mediated processes^14,15,37^, while others seemingly indicate a more direct interaction between ARHGAP25 and the WNT/β-catenin pathway^12,13^. However, we did not find any interaction partners that can be associated with the Wnt/β-catenin pathway, making its relevance less likely in neutrophils.

In conclusion, our study identified 76 potential interaction partners for ARHGAP25 using GST-ARHGAP25 pulldown an co-immunoprecipitation, accompanied by new insights on the biological functions and molecular pathways ARHGAP25 could partake in. As many of the potential partners overlap with former studies on RacGAP interactions, these findings might indicate the existence of several undiscovered territories in GAP physiology. Novel interactions of ARHGAP25–RAC2 and ARHGAP25–RHOG were detected through proteomic and Western Blot analyses, revealing the dependency on the state of the small GTPases for interaction strength. In silico prediction of dimer assembly pinpointed the GAP domain as a credible interaction site, implying the potential for ARHGAP25 to exert its GAP activity on RAC2 and RHOG in the cell. Additionally, we uncovered a previously unrecognized potential regulatory mechanism for ARHGAP25 involving phosphorylation-dependent interaction with 14-3-3 protein family, which might hide an unrecognized regulatory mechanism involving phosphorylation-dependent interaction with 14-3-3 protein family members, which could explain ARHGAP25’s decreased GAP activity upon phosphorylation.

## Materials and methods

### GST-fused recombinant protein preparation

GST and GST-fused ARHGAP25 proteins were produced in One Shot™ BL21 Star™ Escherichia coli bacteria [Thermo Fischer Scientific]. Bacteria were cultured overnight in Lysogeny broth [Sigma-Aldrich] medium (supplemented with 100 µg/mL ampicillin [Merk]). Protein expression was induced by adding 0.5 mM Isopropyl β-d-1-thiogalactopyranoside [Sigma-Aldrich] (37 °C, 3 h). After centrifugation, pellets were lysed by sonication in a lysing solution containing 50 mM Tris (Sigma-Aldrich; pH 7.6), 50 mM NaCl [Biolab Zrt], 5 mM MgCl_2_, and 1 mM dithiothreitol, 1 mM Na-EGTA, 10 μg/mL aprotinin, 10 μM leupeptin, 2 μM pepstatin A and 1 mM phenylmethylsulfonyl fluoride (PMSF) [each purchased from Sigma-Aldrich] added right before use. Debris was separated by centrifugation (8000 g, 20 min, 4 °C), and the supernatant was incubated with Pierce™ Glutathione Agarose beads for 30 min at 4 °C followed by three washes. Protein-coated beads were stored at − 80 °C until further use. Supplementary Fig. S3 provides representative images of the purity and yield of our recombinant proteins and a list of common contaminants detected by MS.

### Isolation of neutrophilic granulocytes and ethics statement

Buffy coat of venous blood was drawn from healthy adult male volunteers according to the ethics permit reviewed by the Committee of Science and Research Ethics of the Medical Research Council (ETT TUKEB) and approved by the Department of Health Administration of the National Public Health Center of Hungary (31937-7/2020/EÜIG). All research was performed following the relevant guidelines and regulations in accordance with the Declaration of Helsinki, and informed consent was obtained from all participants. Neutrophilic granulocytes were separated from other leukocytes by the dextran sedimentation method followed by Ficoll-Paque gradient centrifugation as previously described^2^. After cell counting, aliquots containing 40 million cells were snap-frozen in a pellet form and stored at − 80 °C until further use.

### Cell lysis, GTPγS, and GDPβS loading of cell lysate

Neutrophils were gently suspended in 1 mL diluted cell lysis buffer [Cell Signaling] (supplemented with 1% Protease Inhibitor Cocktail, Phosphatase Inhibitor Cocktail 2, aprotinin [each purchased from Sigma-Aldrich], and PMSF right before use) and lysed on ice for 5 min. The insoluble pellet was removed by centrifugation (12,000 g for 10 min at 4 °C), and the supernatant was equally divided into four portions.

For the active state enrichment of the GTPases, cell lysate was treated with GTPγS [Sigma-Aldrich] by incubating the lysate with 15 mM EDTA [Biocenter] and 20 µM GTPγS for 15 min (RT, 250 rpm), then placed on ice, and MgCl_2_ solution was added at 60 mM final concentration to stop the reaction.

For the inactive state enrichment of the GTPases, cell lysate was treated with GDPβS [Sigma-Aldrich] by incubating the lysate with 15 mM EDTA and 10 mM GDP for 15 min (RT, 250 rpm), then placed on ice and MgCl_2_ solution was added at 60 mM final concentration.

### GST-ARHGAP25 pulldown

GST-ARHGAP25 and GST-coated beads were incubated with intact total cell lysate, GTPγS- or GDPβS-loaded cell lysate for 45 min at 4 °C. After centrifugation (2000 g, 1 min, 4 °C), the supernatant was aspirated, and beads were washed thrice with PBST. Bound proteins were eluted by boiling the beads (at 95 °C for 5 min) in 30 µL 2 × Laemmli buffer. Eluted samples were stored at − 20 °C until measurement.

### Co-immunoprecipitation of endogenous ARHGAP25 from neutrophilic cell lysate

Magnetic beads [Miltenyi] were coated with either polyclonal ARHGAP25 antibody or rabbit IgG and incubated with intact total cell lysate for 45 min at 4 °C. Beads were separated from the supernatant with magnetic force and washed thrice with PBST. Bound proteins were eluted by boiling the beads (at 95 °C for 5 min) in 30 µL 2 × Laemmli buffer. Eluted samples were stored at − 20 °C until measurement.

### Sample preparation and mass spectrometry

Formerly elutedsamples underwent gel-aided sample preparation (GASP)^38^: they were reduced by dithiothreitol (10 mM), alkylated by iodoacetamide (22 mM), digested with trypsin and analyzed in a single run on the mass spectrometer^39^. The digestion mixtures were acidified and transferred to a single-use trapping mini-column (Evotip; 1/8 of the samples) and then analyzed with a data-dependent LC–MS/MS method using an Evosep One Stainless steel emitter (ID 30 µm), Column: Analytical Column NT—30 samples/day; desc: Endurance; stationary phase: C18 AQ; size: 0.15 mm and 150 mm; particle size: 1.9 (LC: 15 SPD) on-line coupled to a linear ion trap-Orbitrap (Orbitrap-Fusion Lumos, Thermo Fisher Scientific) mass spectrometer operating in positive ion mode. Data acquisition was carried out in data-dependent fashion, multiply charged ions were selected in cycle-time from each MS survey scan for ion-trap HCD fragmentation (MS spectra were acquired in the Orbitrap (R = 60,000) while MSMS in the ion-trap). Orbitrap Fusion Lumos Method Summary can be found in Supplementary Table S1.

### Label-free quantification (LFQ) and evaluation of MS data

Raw MS files were converted to .mzML format by MSConvert (version 3.0) and processed using the ‘LFQ-MBR’ workflow of the FragPipe software (FragPipe version 19.1, MSFragger version 3.7, IonQuant version 1.8.10, Philosopher version 4.8.1;^40^. Database search parameters and acceptance criteria for identifications were used as default except for ‘Precursor and fragment mass tolerance,’ which were adjusted to ± 5 ppm and 0.6 Da, respectively. A human proteomic database containing decoys and contaminants was downloaded through the program (date: 27th May 2023.), and the sequence of the GST tag was added manually (Uniprot ID: P08515). IonQuant LFQ was selected with ‘MBR top runs’ set to 25.

The CombinedProtein.tsv file was used for statistical analysis by the Fragpipe Analyst (available at http://fragpipe-analyst.nesvilab.org/) following the creators’ guidelines. The list of detected peptides and identified proteins can be found in Supplementary Table S1 for further exploration. Briefly, the MaxLFQ intensity values were converted to the log_2_ scale. Samples were grouped by conditions and filtered (‘Min percentage of non-missing values globally’ = 0, ‘Min percentage of non-missing values in at least one condition’ = 60). Missing values were imputed using the ‘Missing not At Random’ (MNAR) method, which uses random draws from a left-shifted Gaussian distribution of 1.8 standard deviations apart with a width of 0.3 to generate noise for further analysis. ‘Adjusted *p* value cutoff’ = 0.01, log_2_ fold change (FC) cut-off = 2, Normalisation type: Variance stabilizing normalization. Protein-wise linear models combined with empirical Bayes statistics were used for the differential expression analysis of the pulldown samples. The limma package from the R Bioconductor generated a list of differentially expressed proteins for each pair-wise comparison. A cutoff of the adjusted p value of 0.01 (Benjamini–Hochberg method) and a log_2_ fold change two have been applied to determine significantly regulated proteins in each comparison.

The presence and relative abundance of the formerly established candidates were investigated in co-immunoprecipitated samples. Proteins based on their detection rate in the samples were categorized into three groups (‘low’: < 60%; ‘good’: between 60 and 80%; and ‘excellent’: > 80%), creating a confidence score metric. Proteins were also assigned an enrichment score based on their FC value (‘low’: FC < 0.81 (which is the median FC); and ‘high’: FC > 0.81). Based on these metrics, the candidates were sorted into 4 categories: ‘unlikely’, ‘low credibility’, ‘satisfactory’, and ‘high credibility’. All examined candidates and their corresponding values are available in Supplementary Table S1.

### Hierarchical clustering

Filtered and imputed LFQ values were normalized by Z-score across samples and subjected to hierarchical clustering by the Morpheus software (https://software.broadinstitute.org/morpheus). Both columns and rows were clustered with Euclidean distance measure (linkage method: average).

### Immunoblotting

Eluates of pulldown measurements were also used for immunoblotting. Equal volumes of samples along with ProSieve QuadColor Protein Marker [Lonza] were loaded into 4%–15% (w/v) gradient polyacrylamide gels [Bio-Rad] to perform electrophoresis. Separated proteins are transferred to nitrocellulose membranes [Bio-Rad] and stained with Ponceau solution. After a blocking step for 15 min in EveryBlot blocking buffer [Bio-Rad], membranes were incubated with primer antibodies in 1:1000 dilution overnight at four °C (Anti-SYK [cat no: sc-1240] from Santa Cruz; anti-ACSL1 [cat no: 4047S], anti-LDHA [cat no: 2012S], and anti-RHOG [cat no: E9B7Z] from Cell Signaling, anti-RAC2 [cat no: PA5-29681] from Invitrogen; anti-ARB27A [cat no: MAB7245] from Bio-Techne). Bound antibody was detected with enhanced chemiluminescence using horseradish peroxidase-conjugated anti–rabbit-Ig (from sheep) secondary antibody (GE Healthcare) in 1:5000 dilution (1 h at RT). Ponceau staining was used for loading control. X-ray films were scanned at 1200 DPI resolution, and no digital processing was applied that altered the pixel intensities. The uncropped raw images with corresponding Ponceau stainings are collected in Supplementary Fig. S2.

### Functional enrichment

Protein of the satisfactory and the high confidence groups were used to perform functional enrichment analysis using the shinyGO: web server v. 0.80^41^. User threshold was defined by 0.01, and only the “Gene Ontology (GO) biological process” and “Reactome” data source was used for over-representation analysis filtered by term size (min:5, max:1000). Neutrophil-specific background of human origin was used to eliminate false positive results^42^. Corresponding data is available in Supplementary Table S4.

### STRING analysis

The STRING platform v. 12.0^43^ was employed to collect all documented physical interactions between our potential protein partners identified by MS. Our focus was directed explicitly towards experimentally determined interactions and databases, which meant excluding the software's data mining option to ensure a more robust network acquisition. The program assigned each interaction a score from 0 to 1, corresponding to the strength of evidence supporting the interaction. A cut-off value of 0.400 was applied to filter out less probable interactions. Proteins with no identified interaction were excluded. The obtained network was imported to Cytoscape v. 3.10.1.^44^ for data visualization. Physical interactions are represented by edges between proteins, denoted by nodes.

### In silico PPI prediction by Colabfold and Neurosnap

Direct interaction formation of ARHGAP25, its truncated sequences, and potential partners was predicted in silico by AlphaFold optimized for multimers^16,17^. The main settings were as follows:

num_relax: 0

template_mode: none.

msa_mode: mmseqs2_uniref_env.

pair_mode: paired.

model_type: AlphaFold2_multimer_v3.

num_recycles: 20.

recycle_early_stop_tolerance: 0.5

relax_max_iterations: 200.

pairing strategy: greedy.

max_msa: auto.

num_seeds: 2

Dimers with fewer than 500 AAs were generated with ColabFold v1.5.5^45^. In contrast, any multimer with a total number of AAs over 500 was run by Neurosnap Inc. (Computational Biology Platform for Research. Wilmington, DE, 2022. https://neurosnap.ai/.) because of heavy computational labor. Results are available in Supplementary Table S3.

### In silico prediction of 14-3-3-binding phosphosites

We employed an improved prediction web interface created by Madeira et al., following the Authors’ guidelines^19^. The algorithm scores each phosphosite according to three different criteria and highlights the ones with a high probability of being a binding site for 14-3-3 proteins. Results are available in Supplementary Table S3.

### Data presentation and statistical analysis

Plots were created with GraphPad Prism v. 10.2.0. The predicted dimer was visualized in ChimeraX, and Fig. 2d was created with Cytoscape v. 3.10.1. Each statistical test and corresponding criteria for significance were chosen based on the properties of the data and described in detail in the adequate method section and figure legend.

### Supplementary Information

Supplementary Figures.

Supplementary Table S1.

Supplementary Table S2.

Supplementary Table S3.

Supplementary Table S4.

## Data Availability

Mass spectrometric raw data is available at Figshare (DOI: 10.6084/m9.figshare.25296223; 10.6084/m9.figshare.26303803) and data reported by the FragPipe and the FragPipe-Analyst software are available in Supplementary Table S1.
