# Supplementary material for: Neutrophil-specific interactome of ARHGAP25 reveals novel partners and regulatory insights
Source: Sci Rep. 2024 Aug 29;14:20106. doi: 10.1038/s41598-024-71002-4 (PMC11362597; doi:10.1038/s41598-024-71002-4)
Supplement: Supplementary file 1 — Supplementary Figures. [file 41598_2024_71002_MOESM1_ESM.pdf]

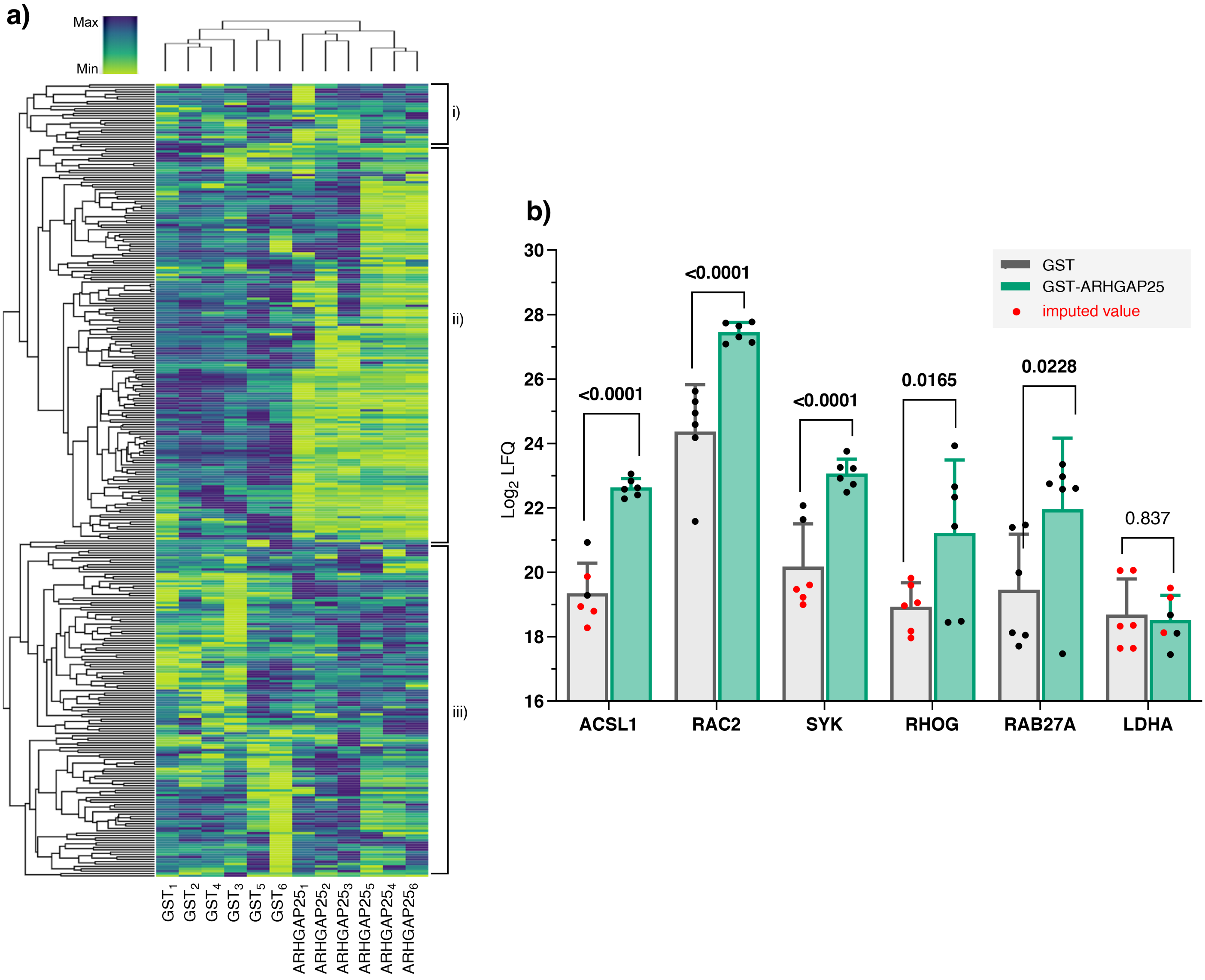

Supplementary Figure S1 - Relative protein abundances among the GST-pulldown samples Complementary figures to investigate the proteomic results of the GST-pulldowns. a) Hierarchical clustering of samples (columns) and proteins (rows) after Z-score normalization, shown as a heatmap. b) Comparison of LFQ values of proteins selected for Western Blot analysis (n=6). Data are presented as mean ± SD. Adjusted p values (extracted from Figure 2/a) are presented numerically; bold values are considered significant (<0.05). Imputed LFQ values are shown in red.

Supplementary Figure S2

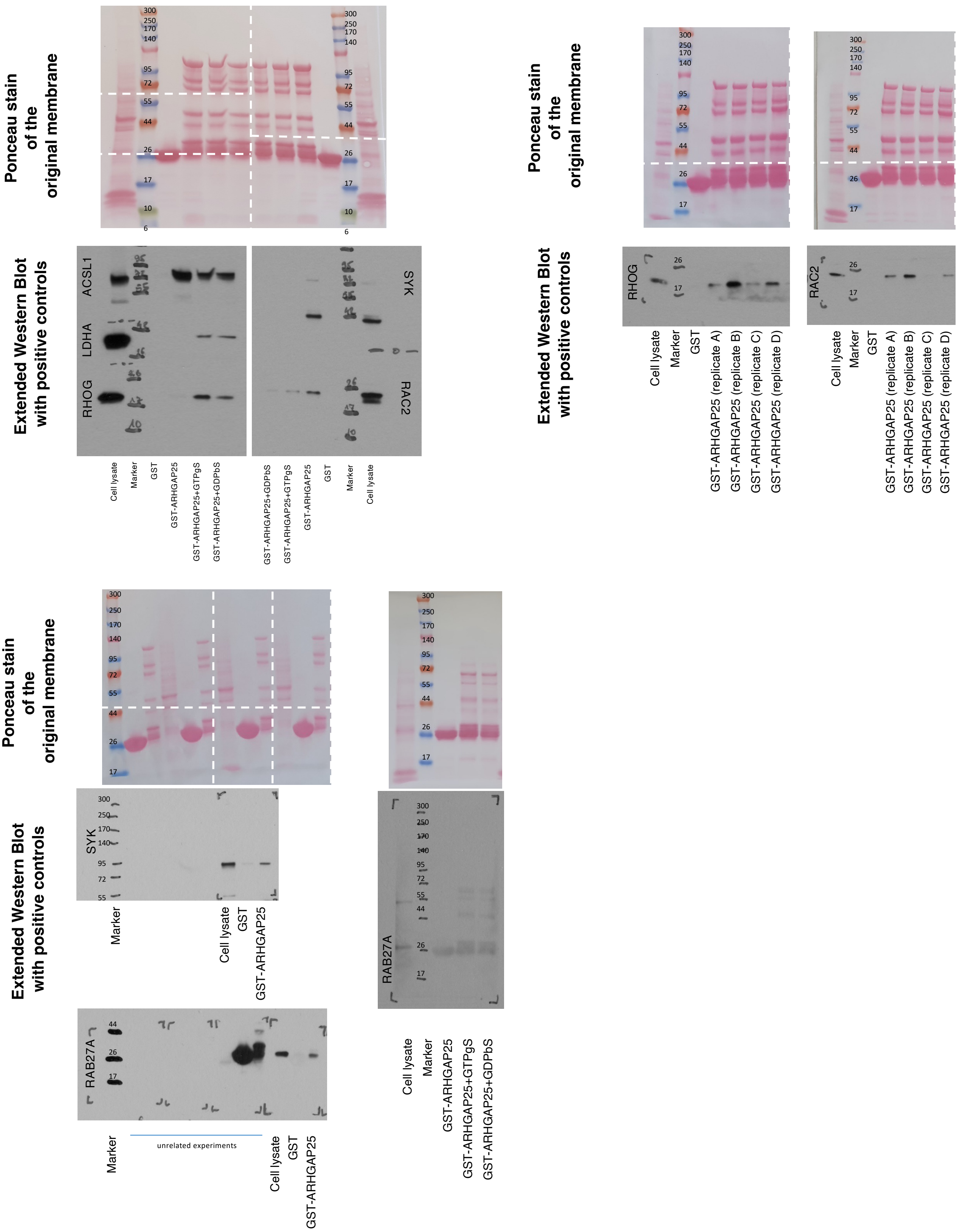

Supplementary Figure S2 – Collection of raw Western blot and Ponceau-stained membrane images. All images used for representation in Fig. 2 and 3, along with additional RAC2 and RHOG detection experiments, are presented here, with corresponding Ponceau-stained membrane images.

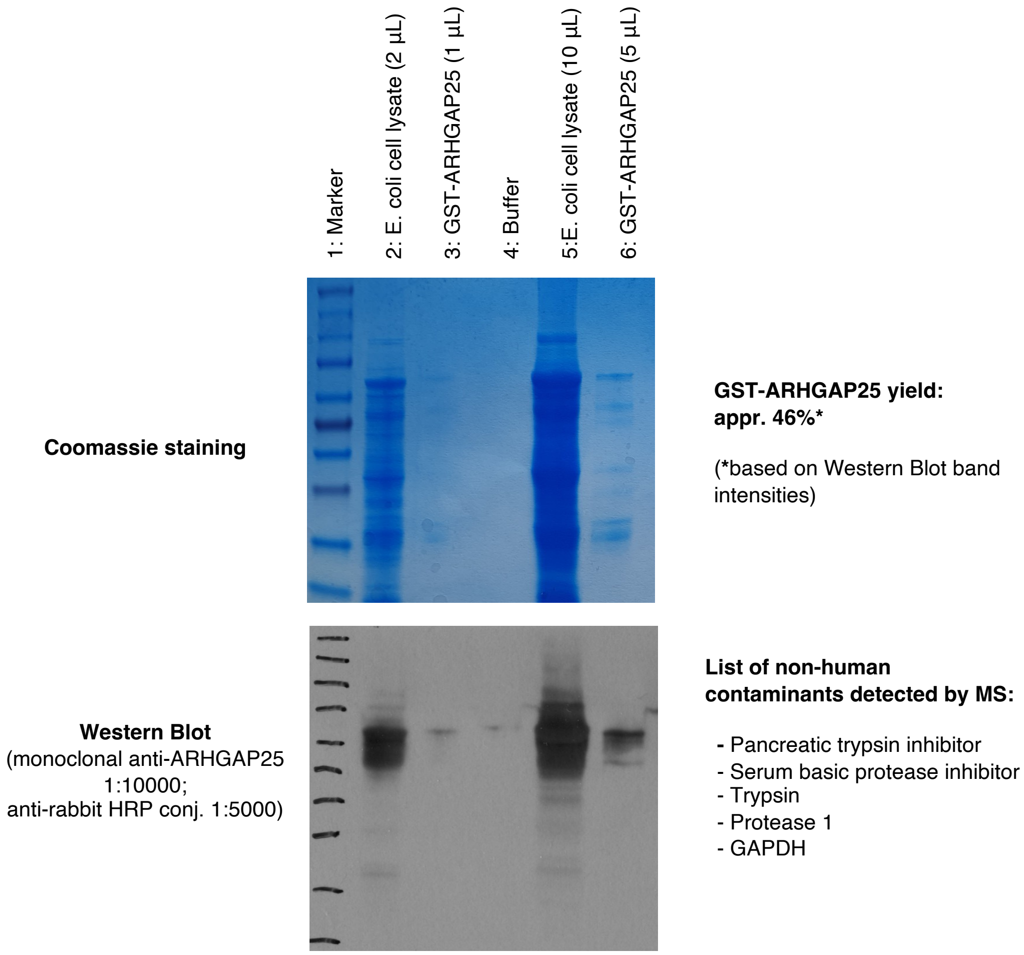

Supplementary Figure S3 – Evaluation of the bacterial expression and subsequent isolation of GST-ARHGAP25. The bacterial production and isolation of GST-tagged ARHGAP25 is monitored by sampling the bacterial cell lysate and the purified recombinant protein. The purity of the samples and abundance of ARHGAP25 were evaluated on Coomassie-stained gels and Western blot images. The yield is calculated based on the band intensities of the developed X-ray films against ARHGAP25, corrected for the initial volumes and dilution of the samples. The list of contaminants in the GST-ARHGAP25 eluates identified by MS is listed.
